# Mixed-methods evaluation of how a predictive model pilot intervention addresses patient non-attendance at outpatient services in an NHS Foundation Trust in England

**DOI:** 10.1136/bmjopen-2025-102154

**Published:** 2025-12-18

**Authors:** Louise Laverty, Amy McCawley, Norina Gasteiger, Thomas Jones, Anthony Wilson, Sam Evans, David Jenkins, Dawn Dowding

**Affiliations:** 1 School of Dentistry, University of Leeds Faculty of Medicine and Health, Leeds, UK; 2 Manchester University NHS Foundation Trust, Manchester, England, UK; 3 School of Health Sciences, University of Manchester, Manchester, UK; 4 Adult Critical Care, Manchester University Hospitals NHS Foundation Trust, Manchester, UK; 5 Division of Informatics, Imaging and Data Sciences, The University of Manchester, Manchester, UK

**Keywords:** Digital Technology, Artificial Intelligence, Health Services Accessibility, Health Workforce, Health policy

## Abstract

**Background:**

There is interest in using predictive models to address non-attendance of healthcare appointments without prior notification. Although several National Health Service (NHS) hospital trusts have piloted predictive models for non-attendance, there is a lack of published evidence in clinical settings.

**Objectives:**

This mixed-methods evaluation of the pilot of a predictive model intervention in outpatient services aimed to examine (1) the effect of the intervention on patient non-attendance and (2) staff engagement in the delivery of the intervention.

**Design:**

A mixed-methods study across two pilot phases. Quantitative data explored the use and impact of the predictive model on non-attendance. Z-tests were conducted to assess changes to non-attendance rates prepilot and in the two phases. Qualitative ethnographic work included 30 periods of observation and interviews with staff.

**Setting and participants:**

Nine outpatient services in an NHS Foundation Trust that volunteered to pilot the predictive model intervention. Qualitative participants were NHS clerical and administrative staff delivering the intervention and service managers.

**Intervention:**

An off-the-shelf predictive model, consisting of a cloud-based, random forest algorithm, produced a risk score of non-attendance by drawing on information from patients’ electronic health records. Staff in the pilot sites attempted to phone patients with a risk score to remind them of upcoming appointments.

**Results:**

Quantitative analysis showed that in phase 1, there were low volumes of intervention calls made across services, but three of nine outpatient services significantly reduced their non-attendance rate. There was a lower overall call rate in phase 2 among the four remaining participating services. One significantly reduced its non-attendance rate from 20.4% to 19.1% (p<0.05). Thematic analysis of the qualitative data suggests that many staff delivering the intervention struggled with actioning the intervention workflow, especially when it was not seen to fall within their defined roles. Delivering the intervention exposed unanticipated operational issues around access provision and communication. Differing perceptions of how the intervention should be prioritised, especially in the context of long waiting lists and staff resourcing issues, offer some context to the limits of the pilot’s success.

**Conclusion:**

The predictive model intervention was positioned as a simple solution to address a complex problem; however, there were challenges inherent in deployment within a dynamic healthcare setting. The sustainability of the intervention and its impact on patient experience warrants further exploration.

**STRENGTHS AND LIMITATIONS OF THIS STUDY**

- This is the first mixed-method study to examine the use of a predictive model for non-attendance in clinical settings. This collaborative study pulls together quantitative analysis from electronic health records with insights from qualitative observations of services, strategic meetings and stakeholder interviews to give a comprehensive insight into the pilot.
- Nine diverse services were involved in the pilot, including services for older patients, women of reproductive age and those with various and comorbid health conditions.
- As an applied health research study implemented in a ‘live’ health system environment, there are many confounders (including other ongoing activities to reduce non-attendance) which may have impacted the success of the intervention.
- There was variation in the uptake and delivery of the intervention.

## Background

 Demands on healthcare systems have increased exponentially over the past decade. The pressures of managing ageing populations, chronic and comorbid health conditions, rising healthcare costs and workforce shortages are commonly reported contributors across nations. Governments are looking for strategies to manage resources better, such as digital technology. The greater investment in digital infrastructure and technologies, which promise to tackle many of these challenges by freeing up clinicians’ time, allowing for more effective allocation of resources and improving patient engagement and care, is then a logical strategy.^1 2^ In the UK, successive governments have published policies stating that digital technologies are essential to the future of the National Health Service (NHS).^3^,^6^ To date, however, studies examining the adoption of digital tools into complex clinical environments show that efforts are often fraught, with high rates of non-adoption.^7^ Nevertheless, implementation efforts continue, with an observed trend of digital tools entering clinical practice through less formal routes, such as living labs and pilots.^8 9^ Some pilots may result in system-scale adoption, but their results are rarely published in the literature.

Risk-stratification predictive models (hereafter referred to as predictive models), algorithms developed to generate individual risk scores for anticipated resource use, are an example of a digital tool intended to address the problem of resource allocation in health systems.^10^ Predictive models are often used to develop an anticipatory care pathway, where an intervention is delivered to those identified as being at high risk.^11 12^ As healthcare organisations undergo digital transformation, predictive models utilising the availability of large data sets from electronic health systems have gained popularity. The evidence on their efficacy and implementation, however, is contested. In the USA, concerns have been raised about how these algorithms can reproduce bias and inequalities through the data used to train them.^13 14^ A recent review of predictive models in primary care cautions that there is insufficient evidence of their clinical benefits to justify their introduction.^11^ The authors highlight that there is currently little evidence about how best to use the outputs of the model.^11^

One area where predictive models are being increasingly developed and introduced in healthcare settings is around patient non-attendance. In the UK, in the context of long waiting lists, addressing non-attendance at healthcare appointments without prior notification (also referred to as did-not-attends (DNAs)) is a key priority. Research suggests that there are often complex patient- and system-level reasons for non-attendance, which reflect inequalities in access to healthcare.^15^,^17^ Interventions to address non-attendance range from charging non-attendance fees,^18^,^20^ overbooking clinics^21^ or telephone calls/text message reminders.^22^,^24^ A rapid review of the effectiveness of these interventions on non-attendance showed limited evidence for their acceptability and cost-effectiveness.^23^ Lindsay and colleagues^16^ propose that previous interventions to tackle non-attendance have had limited success as they fail to distinguish between those who miss single appointments and those who miss multiple appointments. They suggest that groups missing multiple appointments are a specific group who are more likely to be economically deprived, have chronic health conditions and have higher rates of mortality, requiring different support for attendance.^25^,^27^

There are several NHS hospital trusts that have piloted predictive models for patient non-attendance over the past few years, but the findings are limited to reports in the grey literature.^28 29^ Given the lack of clarity in the evidence base, there is a need to understand how predictive models for non-attendances are implemented in real-world settings. This mixed-methods study includes both an analysis of routinely collected NHS Trust data and qualitative observations and interviews of the predictive model intervention for patient non-attendance as it is piloted in outpatient services at an NHS hospital trust. This study aims to (1) examine the effect of the predictive model intervention as it is introduced in some clinical outpatient services and (2) understand how staff engage with and deliver the associated intervention to manage no-shows in outpatient appointments.

## Methods

### Approach and ethics

The mixed-methods evaluation was a collaborative effort, with data analysts from the NHS Trust providing quantitative data on live data from the electronic health system alongside qualitative work to explore how it was being used in practice. Initial findings from both parts of the study were presented at weekly reporting groups, with insights informing the ongoing support to services.

The study was reported using the Good Reporting of a Mixed Methods Study checklist.^30^ The qualitative work was approved by the Health Research Authority (HRA) (NHS Research Ethics Committee Reference: 23/HRA/1259). The quantitative work was exempt as it was part of the service evaluation conducted by the Trust for the pilot. All procedures were conducted in accordance with the ethical standards of the 1964 Helsinki Declaration and followed NHS Health Research Authority guidelines.

Data were collected during a pilot of the intervention in the NHS Trust running from March to September 2024 and included two distinct phases:

- Phase 1 from 25 March to 17 June 2024 with nine outpatient services.
- Phase 2 from 12 August to 1 November 2024 with four outpatient services.

#### Description of the intervention

The NHS Trust purchased an off-the-shelf Risk of Patient No‐Show predictive model from a US-based provider; as such, the model selection was based on convenience. The model consisted of a cloud‐based, random forest algorithm, initially developed, trained and calibrated in the USA. The model weights were then tuned on around 1.5 years of local data from the NHS Trust. The model predicted a patient’s likelihood of not attending an appointment by drawing on information from a patient’s electronic health record (EHR). The model drew on variables covering appointment characteristics and the appointment history to produce a numerical risk score between 0 and 1. The supplier, in response to concerns in the USA about potential bias, had removed protected characteristics (eg, sex, ethnicity) included in earlier iterations from the model.

Administrative and clerical staff generated a confirmation scheduling report on the electronic patient record system. In the report, each patient was assigned a risk score, and this was sorted into groups indicating the likelihood of non-attendance (low, medium and high risk). The cut-offs were based on a review of early results and the proportion of patients within the different score bandings to ensure that an appropriate number of high-risk patients were displayed. Staff made phone calls to patients, prioritising high-risk groups. The purpose of the calls was to confirm whether a patient was intending to attend their appointment and to offer any support required (eg, transport or interpretation services). The script is presented in the online supplemental file.

### Patient and public involvement

At the start of the study, an organisational Patient and Public Involvement panel was involved in the design, priority setting and topic guide production. Two patient and public involvement members attended monthly update meetings and gave feedback on progress, analysis and dissemination plans for the project.

#### Setting, sample and recruitment

This study draws on qualitative and quantitative data from large acute NHS Trusts. The secondary care provider in England comprises 10 hospitals located on four sites and two sizeable local care organisations, providing care for the entire lifecycle of an approximately 850 000-person population. The NHS Trust delivers services that include highly specialist medical services, urgent and emergency care, outpatient and community services, and mental health services for children and young people.

The research plan was presented for feedback at a Trust strategic meeting in June 2023. In February 2024, the implementation lead invited clinical services across the four sites of the Trust to volunteer to pilot the tool and intervention. Nine services in phase 1 initially volunteered, and the operational leads of these clinical services consented to participate in the research evaluation. In phase 2, five services participated in the second pilot to take part, but due to team changes, one dropped out. Three of the four services had taken part in phase 1. The other was new. It is important to note that all pilot services were configured differently in terms of management, staffing and procedures. Some services were also running other outpatient activities to reduce non-attendance, such as patient-initiated follow-ups, two-way text reminders and fast-pass systems.

Individual staff in clinical services who were responsible for managing the pilot workflow and delivering the intervention (eg, Medical Secretaries, Clinic Coordinators, Clerical Officers and Administrative Clerks) were asked to consent to observations. They were sent introduction emails from the researcher, or appropriate manager, with the information sheet and link to an online consent form hosted on the password-protected site Qualtrics. Senior leaders, identified through attendance at strategic meetings, were also invited to participate in interviews and provided online consent. Informed consent was given before all observations and interviews.

### Data collection

#### Quantitative data collection

System data on the intervention delivery were extracted for analysis. This included outputs from 29 406 calls across both pilot phases. There were a total of 25 847 appointments with a call, meaning that some patients received multiple calls. Data quality issues were corrected before analysis. As the predictive model was an off-the-shelf product, the evaluation did not analyse the accuracy or validity of the model outputs.

#### Qualitative data collection

The qualitative data were collected as part of a longitudinal evaluation. This paper draws on ethnographic observations and interviews of staff in clinical services piloting the intervention between March and September 2024. The evaluation also included the collection of documentary evidence (reports, minutes of implementation meetings) and semistructured interviews with senior leaders, but they are not reported here.

Three researchers, two academic (LL and NG) and one clinical (AM), collected the observational data. Thirty field notes were made between the researchers. The researchers used a proforma containing the observation template and topic guide (see online supplemental material) to record observations of staff managing the predictive model workflow and delivering the intervention and conducting ethnographic interviews. The researchers asked staff about their background and perception of non-attendance in the service. They then observed the staff member delivering the intervention and asked questions if clarification was required to understand the decisions that were made. Observations were conducted on-site in person at each clinical service, when practical considering staff workload and availability. Field notes were typed up on leaving the site and stored in a secure data storage environment.

### Analysis

#### Quantitative

Data were extracted for all pilot services for appointments, along with the relevant call and model prediction data. Baseline data were taken for the 6 months prior to the pilot start date and the full duration of the pilot itself. An additional analysis exploring the impact on non-attendance by risk groups could be conducted for the new service in phase 2, as non-attendance scores could be generated both prepilot and postpilot.

R was used to analyse and process the data. Non-attendance rates were compared prepilot and in the two pilot phases, and Z-tests were carried out to assess statistical significance. For changes in the non-attendance rate, statistical significance was calculated using a one-sided z-test with a 5% significance level, on the assumption that the intervention would not increase the non-attendance rate.

#### Qualitative

Qualitative data were anonymised before being managed in NVivo. Thematic analysis was used to analyse the materials systematically.^31^ The approach to analysis was mostly inductive and drew on sociotechnical and ethnographic principles.^32^ The sociotechnical approach moves away from simplistic conceptions of technology as driving organisational change to explore the close interplay between technological, organisational and social dimensions in shaping the design and implementation of technological systems and their organisational outcomes. One researcher (LL) first conducted line-by-line coding and sorted the codes into topic-related first-order codes. A second researcher (NG) reviewed these codes and both researchers then independently refined them into second-order codes. Three researchers (LL, NG and AM) then met to discuss them to develop themes and understand how they relate to each other. Representative quotes for each theme are presented in the paper, with additional quotes available in the supplementary files.

## Results

### Intervention uptake and impact on non-attendance rates

System data show that services were not making high levels of calls. The volume of calls during phase 1 ranged from 3% to 39% of potential patients in the different services, while in phase 2, this ranged from 8% to 34%. Among the nine services in phase 1, three had statistically significant reductions in their non-attendance rate (see table 1). One confounding variable, however, was that all three of these services were also engaged in other activities to reduce non-attendance rates, making it challenging to determine whether it was the intervention alone that was effective.

**Table 1:** Percentage of patients called and non-attendance rates before the pilot and after phase 1

Services	Prepilot non-attendance rate (%)	Phase 1 calls (%)	Phase 1 total calls	Phase 1 non-attendance rate (%)	P value
Service 1	11.5	39	990	11.1	0.31
Service 2	9.4	15	131	9.6	0.54
Service 3	9.7	22	858	10.1	0.7
Service 4	10.5	16	947	9.4	0.01*
Service 5	9.2	25	3167	8.9	0.19
Service 6	17.1	17	543	16.9	0.39
Service 7	10.0	39	1980	7.2	0.00*
Service 8	11.3	3	1190	10.9	0.01*
Service 9	20.4	14	942	22.2	1.00

* denotes p<0.05. Significance should be interpreted with caution due to the ‘live’ operational environment in which the intervention was implemented and a potentially underpowered sample size.

There was a lower overall call rate in Phase two among the four services, and only one service was able to significantly reduce its non-attendance rate (see table 2).

**Table 2:** Percentage of patients called and non-attendance rates before the pilot and after phase 2

Services	Prepilot non-attendance rate (%)	Phase 2 call rate (%)	Phase 2 total calls	Phase 2 non-attendance rate (%)	P value
Service 5	9.2	29	2423	9.7	0.94
Service 7	10.0	8	243	10.3	0.68
Service 9	20.4	34	1347	19.1	0.05*
Service 10	13.3	27	857	14.1	0.31

* denotes p<0.05. Significance should be interpreted with caution due to the ‘live’ operational environment in which the intervention was implemented and a potentially underpowered sample size.

### Impact on non-attendance by risk group

A new service (service 10) took part in phase 2, enabling exploration of the interventions’ impact on non-attendance by risk group. As the service had used the model since August, the data are split pre/post-August, with data extracted until 1 November 2024.

Approximately 60% of patients in this service were in scope for a confirmation call. Of these, only around 40% were called. A significant reduction in the non-attendance rate (p<0.05) was only observed in the medium risk score band (see table 3).

**Table 3:** Non-attendance and call rates by predictive model risk score bands for service 10

Risk score band	Prepilot non-attendance rate (%)	Pilot call rate (%)	Pilot non-attendance rate (%)	P value
Low	4.6	15	7.1	0.97
Medium	16.2	29	13.8	0.05*
High	32.4	53	30.7	0.14

* denotes p<0.05. Significance should be interpreted with caution due to the ‘live’ operational environment in which the intervention was implemented and a potentially underpowered sample size.

### Qualitative findings

The qualitative findings report on what happened on the ground during the two phases of piloting and give context to the quantitative results. The first two themes cover how staff managed the intervention delivery and offer some explanations for the low levels of calls and the variability in effectiveness observed across the different services. The third theme explores unanticipated operational gaps that became evident during the delivery of the intervention and that would not have been picked up in the quantitative analysis. The last theme covers how the intervention was perceived in services and the various adaptations services made to try to sustain delivery.

1. **Thrown in at the deep end: assumptions about staff delivery**

This theme covers how the administrative and clerical staff made sense of the intervention workflows they were told to deliver and how this may have impacted the volume of calls made. The implementation team provided online training, standard operating procedures (SOP) and a telephone script to the administrative and clerical staff; however, this brought to light several gaps and assumptions. First, the hour drop-in training offered for the pilot focused on completing the new digital workflow built into the EHR, assuming that this staff group could confidently use the EHR system introduced 2 years prior. However, observations exposed significant disparities in digital literacy. Over the first week of the pilot, most staff struggled with using the new workflow, leading to a series of data recording errors. The number of errors reduced as the pilot progressed, but staff lacking in digital skills found that the new workflow was a time and effort burden that led to workarounds (digital and non-digital) and avoidance:

The staff member is not sure how to use the workflow. They are unsure what to press when a patient doesn’t answer, so they don’t click anything for all patients, and then they don’t disappear from the work list. The staff member makes notes on a scrap of paper to remember details rather than put them in the system.

Field note 1 (phase 1, week 1, service 3)

Second, the SOP and telephone script offered standardisation among the different services to monitor and compare the pilot’s progress. At the same time, services were also encouraged to adapt these procedures to local practices and needs (contingency). However, this tension between standardisation and contingency led to a lack of clarity for services. Pilot staff frequently were unclear about which groups to call (high risk or everyone), when to call, or how many times to call. The decisions were often left to the discretion of managers who themselves were unclear and did not have the time to engage with guidance. Instead, service demands shaped these decision-making processes, meaning that there were instances of patients being called too far in advance (who would have confirmed through other means) or too late to reschedule an appointment:

Staff are just calling the day before the appointment. They were calling a week earlier, but they got too behind on other work, so they decided to change the timeframe.

Field note 2 (phase 1, week 5, service 5)

After reviewing the data and receiving feedback from the first round, the implementation team gave more formalised procedures for services and staff. For example, the data analyst recommended calling 3 days before an appointment as optimum, giving patients enough time to respond to text or app reminders and giving staff enough time to manage any actions from the calls. This information, however, did not always seem to trickle down to the staff on the ground.

1. **Making it work: delivering the intervention as taken-for-granted work for staff**

This theme looks at how delivering phone calls to this population was taken for granted as part of the role of administrative and clerical staff. While the quantitative data provide evidence about the volume of calls made, the qualitative approach illuminates the factors which may have impacted the quality of calls and limited the impact of the intervention. The business case for the pilot described the intervention as a phone reminder by staff who were already contacting patients and, as such, did not anticipate it requiring further resources. In some pilot services, staff who regularly called patients to remind them of appointments saw the intervention as an amendment to an existing practice rather than a new one. They used the workflow lists and made the reminder phone calls as they were accustomed to. The script for the intervention calls, however, did include additional questions to encourage engagement and to ask if patients required any additional support to attend appointments. Most of the staff in the pilot services did not access or use the script. Some were very vocal about not wanting to follow a telephone script, which felt unfamiliar and ‘corporate’, and that disregarded their expertise and experience of contacting patients:

The service manager reports that the staff were not keen on scripts and felt they could make calls their own way.

Field note 3 (phase 1, week 3, service 4)

The pressures on staffing in services meant that the pilot work was sometimes delegated to other administrative and clerical staff, such as ward clerks, medical secretaries and care navigators, who did not regularly call patients. These staff members often found the work unfamiliar and, in some cases, uncomfortable and undesirable:

One staff member was very unhappy about the work and said they hadn’t signed up to work in a call centre. They call in sick the following day.

Field note 4 (phase 1, week 4, service 4)

It was noted across the observations of the first pilot that most phone calls were quick, closed, reminder calls, hinting that staff were not as comfortable making reminder calls as anticipated. Staff were not following the script, nor were they offering any additional support. Typically, calls were in the form of statements—‘I’m just calling to remind you of your appointment on this date in this department’, or closed questions—‘I’m calling to check you are still coming to your appointment on this date?’. This meant that phone calls could be completed quickly, sometimes taking as little as 20 s, which was necessary given the demands on staff, but did not feature the engagement and offers of support anticipated by the intervention leads.

1. **Intervention delivery exposing operational gaps**

This theme highlights important operational gaps that were made visible during the intervention delivery, importantly around accessibility and patient communication, and that would not have been evident in the quantitative analysis alone.

- **Unfamiliarity with accessibility procedures**

There were some instances where the intervention phone calls did lead to some patients requesting further support. However, during the first pilot, many of the staff, including managers, were unfamiliar with the processes of booking interpreters or transport, leading to protracted discussions about what they could and could not offer:

The patient asks if the hospital can provide transport for them. Staff is unsure about procedures and say they will have to call them back when they’ve spoken to the manager. The manager tells the staff that the patient has to arrange themselves or call the team back and rearrange the appointment.

Field note 5 (phase 1, week 1, service 2)

Many of the staff also struggled when the patient they called did not speak English as a first language. Some staff, not having clarity from the procedures, overtly avoided calling these patients, stating that ‘they assume someone else will call them’ (field note 6, phase 1, week 2, service 9). All three observers noted that some staff appeared to be covertly avoiding calling these patients—they did not work down the call list systematically but would appear to pick and choose which patients to avoid, such as names that were not obviously British. When staff did make these calls, they often resorted to speaking slowly and loudly or trying to speak to a family member:

Staff member says they would always call patients even if a note said they don’t speak English. Will then tell them they’re going to call their next of kin.

Field note 7 (phase 1, week 5, service 5)

Staff also struggled when calling patients who were deaf or hard of hearing (again speaking louder and slowly), had additional learning needs, or were in a different setting (such as a care home or prison). Staff were unsure about their clinical service policies around working with more complex patients and often seemed stressed during calls:

Staff called a patient but the call was answered by a receptionist. Confusion about who the receptionist was. The staff member checked the patient form and saw the name of an organisation. Googled this to find out it was a care home. In the meantime, the patient was put on the phone and confirmed they would attend the appointment. The staff member got visibly frustrated as they aren’t allowed to speak with patients in care homes.

Field note 8 (phase 1, week 2, service 9)

Not all staff groups involved in the pilot could action the outcomes of the call. For example, some administrative and clerical staff could not rebook patients in the system, which meant they had to pass on requests to the booking team, who would contact the patient to reschedule. These staff felt that because they could not action this work, they were not the right group to be doing this:

The staff member reports that they don’t know the system for booking appointments or interpreters, so it takes a long time to ask for help from managers. They suggest the booking team would be much quicker, but they won’t offer to help.

Field note 9 (phase 1, week 1, service 3)

- **Patient confusion over communication**

The observations captured some patient responses to the phone call and asked the pilot staff for feedback on patient responses. One service, in particular, reported high levels of patient frustration with the reminder phone calls, with some patients reporting that they had already confirmed by other means. The service had a two-way text reminder system that allowed patients to reply if they could no longer attend. Staff, however, were not aware whether the system was connected to the patient notes, leading to duplication:

Patients sometimes get frustrated if they have already confirmed via the app. Others get confused as to why they’re being called again.

Field note 10 (phase 2, week 2, service 5)

Most commonly, the reminder phone calls gave patients the opportunity to cancel or reschedule appointments. This highlighted a much broader system issue around cancellations and rebooking procedures for patients in the Trust. Patients often stated that they had tried to cancel their appointment on the phone or by email but that these attempts had not been successful.

The patient says they are abroad and tried to reschedule. Staff says they may not have gotten the message from the receptionist.

Field note 11 (phase 1, week 6, service 1)

1. **Perceived value and priorities of the intervention within clinical teams**

This theme examines how staff and managers perceived the value and sustainability of delivering the intervention. Some managers and staff gave positive feedback about the pilot intervention. Staff who felt the work could be efficiently integrated into their usual remit saw it positively and found the work manageable. Some managers found that the reporting tool was useful operationally, enabling them to backfill cancelled appointments and to evidence their team’s work to reduce non-attendance to senior leaders. Changes in the weekly non-attendance rates were also given as evidence of success:

Think it’s working and helping to reduce DNAs. The DNA rate had gone up when the staff member was on leave and down again when she was making the calls. Thought it was brilliant that it’s working.

Fieldwork interview (service lead, service 9)

Like introducing most interventions in the healthcare system, resourcing was problematic. During the first pilot, two services stopped within the first few weeks due to staff sickness and lack of cover. Among other services, managers were regularly looking to move the work, such as to bank staff or a centralised booking team to protect their staff time. In the second phase of the pilot, only four of the nine services continued in some form, but how this was delivered varied. In one service, a single staff member continued doing all the work for both pilots, which meant the intervention did not happen when they were off work. In the second service, the work was moved to a different staffing group that was not engaged and unable to reschedule appointments. In the third service, calls were made sporadically depending on staff availability. The fourth service claimed to be continuing but did not respond to requests from the implementation team for updates. Managers and leads commonly reported seeing the intervention as ‘nice to have’ rather than an essential part of their core work:

If staffing was okay, the workload could take place when everyone was in, but there is so much long-term sickness that we haven't been as effective as we could have. We tried to provide as much support as possible, but it was unavoidable. If we had staff, we would do it, but if it was an expectation that we have to do it, it would be too strenuous.

Fieldwork interview (manager, service 7)

## Discussion

The findings of this mixed-method evaluation provide a novel perspective on what happens when predictive analytic tools are introduced into practice. Both the quantitative and qualitative findings demonstrate the difficulty of implementing and evaluating a digital tool in healthcare settings undergoing continual change in service delivery, resources and priorities. The findings echo previous research examining the implementation of digital tools in the NHS, such as digital literacy, leadership and resources.^7 33 34^ The evaluation of the pilot intervention can be summarised in terms of simplifying complexity. First, the predictive model itself simplifies a complex problem. The reasons for non, as the literature shows, are complex, overlapping and often specific to the health condition, population and health service.^15 17 26 27^ The nature of non-attendance is also heterogeneous; the reasons for missing a single appointment are likely to differ from those for missing multiple appointments.^16^

Second, the use of reminder calls simplifies the solution for non-attendance. Research on population-level interventions to respond to high-risk groups identified by predictive models for non-attendance is poor and inconclusive.^11^ In our pilot, reminder phone calls were considered the more straightforward option. They were seen as low-cost, as it was assumed that the intervention could be integrated into the everyday work of non-clinical administrative and clerical staff. They were also low-risk, with a reminder phone call not outside of what patients might expect from an outpatient service. The evaluation, however, showed that the assimilation of this work depended on staff roles, work pressures, digital literacy and confidence in engaging with patients. There was also an underestimation of additional work tasks that would ensue from these phone calls. The ability to deal with accessibility needs and complex patients in the first phase of the pilot was flagged as a concern for improvement, and additional training was put in place.

Third, a reminder phone call is a simplification of the mechanism. The assumption being that reminding patients of their appointments will result in action and prevent non-attendance. The quantitative findings do not support this hypothesis, with only a few services seeing a significant impact on non-attendance rates (and those results are not necessarily attributable to the intervention). Evidence increasingly suggests that tailored, individual approaches are needed that also engage with patients to improve service provision.^16 35^ Finally, the qualitative observations suggest that due to wider system issues, reminder phone calls offered the opportunity for patients to cancel or reschedule appointments rather than supporting those reluctant to attend. This finding is supported by other research showing that personal phone reminders (eg, telephone calls) significantly increase cancellation and rescheduling rates.^15^

Our evaluation suggests that further development of appropriate interventions that address the complexity of the problem, solution, mechanism and system is needed. There was some positive feedback on the intervention from staff and managers. However, it was not considered valuable enough to justify diverting resources away from other ‘core’ activities. This was reflected in the smaller number of participating pilot services during the second pilot stage. Despite the mixed results, the NHS Trust invested in continued access to the predictive algorithm. They did, however, tailor the intervention training and develop a resourcing calculator for services, with the rollout moving to an opt-in approach, rather than rolling it out across all services. A longitudinal evaluation will explore whether staff and services continue to use the predictive model tool and intervention in the long term. Further work to explore patient experiences, which were not a focus of the current study, is needed to explore any needed intervention refinements, particularly on how best to support access for vulnerable groups.

### Strengths and limitations

This study was a collaborative effort drawing on insights from multiple methods, investigators and sources including qualitative data obtained through interviews with stakeholders, observations of work processes and quantitative data pulled from the system. Three qualitative researchers with different backgrounds collected the data. This level of triangulation may enhance the credibility and validity of the findings. The diversity of the services/sites involved in the piloting was also a strength. Services for older patients, women of reproductive age and those with various and comorbid health conditions were included. This strengthens the transferability of the findings.

Limitations relate to the quantitative data and the intervention itself. An existing model was used, although there may have been a better model with more appropriate cut-offs. As an applied health research study implemented in a ‘live’ and complex operational health system environment with many policies, interventions and additional approaches to reducing non-attendance, there are many confounders which may have impacted the success of the intervention, and it was not possible to account for all changes that occurred throughout the pilot timeframe. These include other approaches such as patient-initiated follow-up and fast-pass, both of which were introduced to reduce non-attendance. Phase 2 also overlapped with the summer holidays, a period associated with a higher-than-usual non-attendance rate. Additionally, as evidenced by the qualitative data, there was great variation in the uptake and delivery of the intervention, whereby many staff members were making reminder calls rather than engagement ones. Compliance monitoring data (ie, clicks within the system) cannot distinguish between these. Further, there was inconsistency in how the intervention was delivered between staff, due to training limited to workflow (ie, technical training) as well as a reliance on managers to relay information to and support staff. The presence (or lack thereof) of statistical significance can therefore be taken as a guide only. The sample size may have also been insufficient to achieve adequate statistical power, potentially limiting the ability to detect meaningful effects. The quantitative results should therefore be interpreted with caution.

## Conclusion

The intervention in this project was a simple approach to a complex problem. The mixed-methods evaluation has demonstrated the difficulty of implementing a digital tool in healthcare settings undergoing continual changes in service delivery, resources and priorities. Further adaptations to the intervention are required, as well as an exploration of the sustainability of the intervention and patient experience.

## Supplementary material

10.1136/bmjopen-2025-102154 online supplemental file 1

## Data Availability

Data are available on reasonable request.
