# Supplementary material for: Mixed-methods evaluation of how a predictive model pilot intervention addresses patient non-attendance at outpatient services in an NHS Foundation Trust in England
Source: BMJ Open. 2025 Dec 18;15(12):e102154. doi: 10.1136/bmjopen-2025-102154 (PMC12718563; doi:10.1136/bmjopen-2025-102154)
Supplement: online supplemental file 1 [file bmjopen-15-12-s001.docx]

Supplementary Materials

**1. GRAMMS Reporting Checklist**

| **Reporting item** | **Reported where in manuscript** |
| --- | --- |
| (1) Describe the justification for using a mixed methods approach to the research question | Yes, Methods section |
| (2) Describe the design in terms of the purpose, priority and sequence of methods | Yes, Methods section |
| (3) Describe each method in terms of sampling, data collection and analysis | Yes, Methods section |
| (4) Describe where integration has occurred, how it has occurred and who has participated in it | Yes, Methods and Discussion section |
| (5) Describe any limitation of one method associated with the present of the other method | Not applicable. |
| (6) Describe any insights gained from mixing or integrating methods | Yes, Discussion section |

**2. Script provided to staff to guide the phone calls.**

| **Introduction:** | "Hello, my name is (Your Name). I am calling from (Hospital/Department/Location).  Are you aware you have an appointment booked with us on (Date and Time of Appointment)?  Please can you confirm that you can attend your appointment?  **AIS: Do you have any additional communication or information needs?** |
| --- | --- |
| **Understanding their situation and reaffirming why their Appointment matters to them and the NHS.** | “We know that people have lots going on in their lives and sometimes it’s not easy to make appointments.  So, we are ringing to see if there is anything we can do to help you attend so that you get the healthcare you need.  We also know that sometimes people can’t make it and if so, we can free up that appointment slot for someone else – each time we are able to do that it saves the NHS £120.” |
| **Reiterate, You Are There to Help Them:** | "We want to make things as easy as possible for you. Just let us know if getting to the appointment is tricky. We're here to work out solutions together. " |
| **Keeping Them Informed:** | "Please tell us if you need any information or help understanding things. We want to make sure you know everything you need about your appointment." |
| **Ending the Call:** | Your appointment is on (Date and Time).  "Thanks for talking with me today. If you need anything or have questions, please contact us. Have a great day!" |

**3. Observation guide**

Qualitative evaluation of predictive management of outpatient no-shows: Observation Sheet

| Observation number |  | Hospital ID code: |  |
| --- | --- | --- | --- |
| Observation time (hrs) |  | Department ID code: |  |
| Observer initials |  | Number of consented staff being observed: |  |

| **Things to note and ask:**  ***Staff information*** (background, time in service, experience in service, current challenges, if perceive DNA model has role to play in their everyday work); ***Clinic observations*** (waiting times, demographics of patients, DNA rates, perceptions of reasons for DNA); ***Observations of EPR generally*** (staff comments, observations of system in use); ***Observations of DNA model*** (staff accessing report, challenges completing, phone calls, staff responses, any patient responses important to note, needed or wanted changes to work, competing demands, if think can be implemented widely) |
| --- |
|  |

**4. Interview guide**

Note: some edits have been made to the guide to maintain the NHS Trust's confidentiality.

**Semi-structured interview guide: Impact of the predictive model on no-shows**

Note: This guide only represents the main themes that will be discussed with participants. Additional questions or prompts may be asked.

1. **Introduction**

Thank you for agreeing to participate in this study. Our aim is to better understand
how the implementation of the EPR has affected outpatient no-shows in your clinical unit. If you agree, I would like to audio-record the interview to make sure I don’t miss any of your responses.

Do you have any questions about the study or your participation?

Shall I start recording?

1. **Questions**

We are interested in the impact of one component of the EPR, in particular: a predictive model, which shows staff the probability that a patient will miss their upcoming appointment.

● Were you aware that this information has recently become available on the EPR? [if yes: Could you tell me what you think of this feature so far?]

● Do you think that the rate of missed appointments in your unit has changed at all after the predictive model was introduced? [If yes, ask for a description of changes over time.]

● Did the predictive model change the things you do in your unit to prevent or mitigate missed appointments? [Prompt: overbooking, any attempts to contact patients to encourage appointment attendance. If yes, ask which patients the reported strategy is used with, how they are identified, and who/how/when implements this strategy. Ask if they use any additional information in combination with the model prediction, such as information about the patient’s care plan, to decide which strategy to implement.]

o Are there any types of patients or circumstances in which you do not take into account the predictive model’s results?

● Other than your use of [strategy], do you use this information about the patient for any other decisions or actions?

● Did the predictive model affect the way you schedule outpatient appointments? [Prompts: overbooking, is appointment scheduling still managed by the same staff members as before?]

● Did staff receive any training or information before using the model? [If yes: How useful do you think that was? Follow-up: Is there any training (or, if yes: additional training) you think staff may benefit from?]

● Can you think of any other resources that you do not have, which would help you use the predictive model optimally?

● Do colleagues in your unit use the information provided by the predictive model? [Follow-up: How do your colleagues generally perceive the model?]

● Which barriers did your unit face to using the predictive model? [Prompts: patient characteristics, staff’s ability and motivation, unit culture]

● Which factors facilitated the use of the predictive model? [Prompts: patient characteristics, staff’s ability and motivation, unit culture]

● What benefits did you observe from using the predictive model for no-show management in your unit?

o Are there any types of patients or circumstances in which using the predictive model was particularly relevant or helpful?

● Were there any undesirable consequences of using the predictive model for no-show management in your unit?

o Are there any types of patients or circumstances in which displaying the predictive model results may be harmful or inappropriate?

1. **Conclusion**

● Do you have any thoughts on what we discussed that you haven’t had a chance to share thus far?

1. Additional Quotes

| Theme | Related codes | Additional quotes |
| --- | --- | --- |
| 1. Thrown in the deep end: assumptions about staff delivery | Digital literacy, Workarounds  Lack of clarity, guidance, support from manager | The patient confirms, and then the staff member spends considerable time attempting to find out how to confirm in the system. The staff member says she isn’t technical and asks for help with the Excel spreadsheet (unfamiliar with the search and find function and was manually scanning through a large spreadsheet after every phone call). *Fieldnote (Phase 1, Week 1, Service 6)*  There were some issues in setting up the date range to generate the patient lists. The staff member reports that they haven’t been given guidance on when and how often to call patients and ask the manager. The manager says they expect 1.5 -2 weeks to be fine for follow-up appointments. *Fieldnote (Phase 1, Week 2, Service 9)* |
| 1. Making it work: delivering the intervention as taken-for-granted work for staff | Discomfort, issues with the telephone script | The staff member says she finds making the phone calls awkward and finds the script a bit patronising. *Fieldnote (Phase 1, Week 1, Service 2)* |
| 1. Intervention delivery exposing operational gaps | Accessibility, transport, interpreter, support from the manager  Different job roles, actioning outcomes, duplication of work,  Patient response to reminders, duplication of work, discomfort, opportunities for cancellation, communication difficulties | The new manager was not aware of how to determine whether a patient needs transport support, whether they had been assessed for transport and how to actually book this. Said he would look into it. *Fieldnote (Phase 2, Week 3, Service 7)*  The staff member asks the patient if they need an interpreter, and the patient confirms, but the staff member doesn’t know how to book this, especially as the appointment is tomorrow. The manager comes in and tells them to email the request over, and they will try to deal with it. *Fieldnote (Phase 1, Week 1, Service 3)*  The staff member is unsure what to do if the patient doesn’t speak English-needs to go back to the main patient information form to see this. Sometimes a relative will pick up, but they’re not allowed to speak with relatives so unsure how to record this outcome. *Fieldnote (Phase 1, Week 2, Service 7)*  Staff member says it would make more sense for the appointment team to call since they have to do the work anyway. *Fieldnote (Phase 2, Week 3, Service 9)*  Staff said she cannot reschedule over the phone and that she would contact the secretary to do this. Sends a message to the secretary on teams to cancel and reschedule. *Fieldnotes (Phase 2, Week 4, Service 7)*  The staff member is a bit concerned about patients getting indignant. She says that sometimes feels like she is harassing them a bit and sometimes feels uncomfortable. *Fieldnote (Phase 1, Week 3, Service 7 )*  The staff member reports that they often get people asking why they had been called, especially mentioning they had a text reminder. Staff reports that she sometimes tells people they have been flagged on the list as high risk. The response has sometimes been that patients say they have never missed an appointment in their lives. Others ask to be removed from the list. *Fieldnote (Phase 2, Week 3, Service 7)*  The patient says they can’t attend because they can’t get a lift to the hospital. They have tried to call to reschedule but haven’t been able to get through. *Fieldnote (Phase 1, Week 3, Service 9)*  It has shown how hard it has been for patients to get through on the phones. I picked up many cancellations and re-books because they haven't gotten through. Enabled us to backfill some appointments. We want to put direct lines back in but are waiting for the office to finalise. *Fieldwork Interview (Manager, Service 1)* |
| 4. Perceived value and priorities of the intervention within clinical teams | Becoming familiar, fitting into routines, seeing impact, struggles with staffing and resources. | Staff said she likes the system and thinks it could be rolled out more widely. All the information is in one place and the list is all there and you can work your way down. Could become part of daily routine to do the follow-up calls. Said at the start she was a bit hesitant as it’s new but likes it once she got into it. *Fieldnote (Phase 2, Week 1, Service 9)*  Staff member says that their DNA rate is down so they will probably keep going. *Fieldnote (Phase 2, Week 4, Service 7)*  Manager says they are struggling with staffing, high turnover, sickness, vacancies, so have paused taking part in the pilot as having to get their booking team covering reception. *Fieldnote (Phase 1, week 3, Service 6)*  Manager says that as soon as staffing issues come up, this is the first thing to go. It almost feels like a luxury. *Fieldwork* *Interview (Manager, service 9)* |
