# Supplementary material for: Mixed-methods evaluation of how a predictive model pilot intervention addresses patient non-attendance at outpatient services in an NHS Foundation Trust in England
Source: BMJ Open. 2025 Dec 18;15(12):e102154. doi: 10.1136/bmjopen-2025-102154 (PMC12718563; doi:10.1136/bmjopen-2025-102154)
Supplement: Supplementary file 2 [file bmjopen-2025-102154.reviewer_comments.pdf]

PEER REVIEW HISTORY

BMJ Open publishes all reviews undertaken for accepted manuscripts. Reviewers are asked to complete a checklist review form and are provided with free text boxes to elaborate on their assessment. These free text comments are reproduced below.

ARTICLE DETAILS

Title (Provisional)

Mixed-methods evaluation of how a predictive model pilot intervention addresses patient non-attendance at outpatient services in an NHS Foundation Trust in England.

Authors

Laverty, Louise; McCawley, Amy; Gasteiger, Norina; Jones, Thomas; Wilson, Anthony; Evans, Sam; Jenkins, David; Dowding, Dawn

---

VERSION 1 - REVIEW

**Reviewer**	**1**
**Name**	**Rubinger, Luc**
**Affiliation**	**McMaster University Faculty of Health Sciences**
**Date**	**15-Apr-2025**
**COI**	**None**

Consider splitting this study into two papers. Qualitative analysis on adoption of differing call protocol and managing the outcomes of those calls. The second paper could be a more fullsome discussion of the intervention used and the outcomes of the pilot and the data from a more prolonged sustained intervention adoption. this is a qualitative paper and a managerial/MBA style paper blended in one making it confusing and prohibitively long and arduous.

**Reviewer**	**2**
**Name**	**saeed, shazina**
**Affiliation**	**Amity Institute of Public Health**
**Date**	**14-Sep-2025**
**COI**	**None**

Thank you to the editor for the opportunity to review this interesting and relevant manuscript.

This is an important study that addresses a timely issue and adds value to the growing body of evidence in this field. The research question is well-motivated and the study makes a meaningful contribution. That said, I would encourage the authors to consider the following points to strengthen the manuscript:

1)Quantitative methods: Please include more detail on the quantitative methodology used, particularly around model selection, choice of cutoffs, and justification for the statistical tests applied.

2)Confounding variables: Clarify how confounding variables were identified and whether they were adjusted for in the analysis. This will improve transparency and strengthen the validity of the findings.

3)Sustainability issues: It would be useful to elaborate on the sustainability aspects of the intervention, including potential barriers to long-term adoption and any resource implications for scale-up.

4)Recommendations for further research: Provide clearer recommendations that emphasize patient experience and identify specific areas for refining the intervention mechanisms.

VERSION 1 - AUTHOR RESPONSE

Reviewer 1. Suggest splitting the paper into qualitative and quantitative results
- We would like to thank the reviewer for pointing to the disconnect between the qualitative and quantitative sections. As the evaluation was planned and conducted in collaboration as a mixed-method approach, we would like to keep the paper together, especially as the quantitative analysis of the pilot is drawn from system data and, as such, is exploratory and limited in scope. However, to help address these concerns, additional signposting has been included throughout the paper to better integrate the two sections. For example, at the start of page 4 in the methods section, we have added information about the mixed-method evaluation process: "The mixed-methods evaluation was a collaborative effort, with data analysts from the NHS Trust providing quantitative data on live data from the electronic health system alongside qualitative work to explore how it was being used in practice. Initial findings from both parts of the study were presented at weekly reporting groups, with insights informing the ongoing support to services."
- We have also added signposting to the qualitative findings (page 6), describing how each theme gives context and possible explanations to the quantitative results: "The qualitative findings report on what happened on the ground during the two phases of piloting and give context to the quantitative results. The first two themes cover how staff managed the intervention delivery and offer some explanations for the low levels of calls and the variability in effectiveness observed across the different services. The third theme explores unanticipated operational gaps that became evident during the delivery of the intervention, and that would not have been picked up in the quantitative analysis. The last theme covers how the intervention was perceived in services and the various adaptations services made to try to sustain delivery."
- Each theme also has signposting and has been edited to ensure that it is more focused and related to the quantitative findings (see page 7, 8, and 9) to better set the scene.
- We hope that these changes, alongside moving some material to supplementary files,

improve readability.
Reviewer 2: Please provide more about quantitative methods, in particular: Model selection; Choice of cut-offs; Stats justification; managing confounding variables
- We would like to thank the reviewer for pointing out areas where more transparency is needed. We have added the following additional information in the methods section, recognising that the evaluation was taking place in a live clinical environment and decisions do not reflect what may have been planned in a scientific study/trial (as is stated in the discussion and limitation section):
- Model selection: we have confirmed that the model selection was convenience on page 5: "The NHS Trust purchased an off-the-shelf Risk of Patient No-Show predictive model from a US-based provider; as such the model selection was based on convenience."
- Cut offs – we have added the following information on page 5: "In the report, each patient was assigned a risk score, and this was sorted into groups indicating the likelihood of non-attendance (low, medium, and high-risk). The cut-offs were based on reviewing early results and the proportion of patients within the different score bandings to ensure an appropriate number of high-risk patients were displayed"
- Stats – justification page 5: "For changes in the non-attendance rate, statistical significance was calculated using a 1-sided z-test with a 5% significance level, on the assumption that the intervention would not increase the non-attendance rate."
- Confounding variables: Due to the pilot evaluation taking place in a live clinical environment, and that the predictive model did not include demographic information, it is hard to manage and account for all possible confounding variables for the pilot. Instead, we recognise in the text that certain factors (such as differences between clinical services and other ongoing operational activity) may be confounding variables that limit the interpretation of the quantitative findings. However, given that there is so little published on pilots of these interventions in clinical settings, we believe it is important to share findings.
Reviewer 2: Discussion: could you add information about sustainability – barriers to roll out and resource implications
- Additional information has been added to page 11, explaining the ongoing rollout of the intervention within the trust and recognising that the longitudinal evaluation of the rollout will look at sustainability in more depth: "Despite the mixed results, the NHS Trust invested in continued access to the predictive algorithm. They did, however, tailor the intervention training and developed a resourcing calculator for services, with the rollout moving to an opt-in approach, rather than rolling it out across all services. A longitudinal evaluation will explore whether staff and services continue to use the predictive model tool and intervention in the long term."
Reviewer 2: Discussion: recommendations on patient experience, refining intervention dynamics
- We have added information on page 11 around this, reflecting the need for patient experience (which is complex given that many patients would not know that the intervention was a reason behind the phone-call): "Further work to explore patient experiences, which were not a focus of the current study, is needed to explore any needed intervention refinements, particularly on how best to support access for vulnerable groups."

VERSION 2 - REVIEW

**Reviewer**	**2**
**Name**	**saeed, shazina**

Affiliation **Amity Institute of Public Health**

Date **01-Nov-2025**

COI

Thank you for the opportunity to review this paper. It is a well written paper, and the findings are relevant in improving patient engagement, operational efficiency in healthcare settings. The conclusions are well supported by the data. The paper can be accepted in its present from.
